# Let’s Modernize Public Health Care Data

**DOI:** 10.1093/ajcp/aqac156

**Published:** 2023-01-09

**Authors:** Harvey W Kaufman

**Affiliations:** From Quest Diagnostics, Secaucus, NJ, USA

Dr Rochelle P. Walensky, Director of the Centers for Disease Control and Prevention (CDC), has challenged the CDC to be reestablished as the premier public health agency charged with protecting the United States from health, safety, and security threats.^1^ Part of the CDC challenge is to modernize the approach to collect and analyze health care data, including clinical laboratory results. Dr Walensky succinctly stated the need for the CDC to “share scientific findings and data faster.”^1^ The coronavirus disease 2019 (COVID-19) pandemic taught us the value of daily collecting and sharing such data more quickly (ie, information from the prior day’s laboratory testing, reported cases, hospitalizations, deaths, and vaccinations). National severe acute respiratory syndrome coronavirus 2 (SARS-CoV-2) seroprevalence studies of communities, supported by the CDC, provided timely insights that ­seropositivity was far higher than case report data suggested.^2^ Weekly SARS-CoV-2 genotyping data, supported by the CDC, provided national and regional trends in the prevalence of viral variants.^3,4^ Likewise, the CDC provided timely weekly data updates on ­monkeypox that are valuable in tracking disease spread and control.^5^

Often stated, 70% of health care decisions involve clinical laboratory testing. Clinical laboratory data are vital to gain a better epidemiologic understanding of clinically significant conditions from a public health perspective (eg, COVID-19, emerging infections, sexually transmitted infections, pediatric lead poisoning, and numerous other health conditions). The core source of many CDC epidemiologic reports remains the National Health and Nutrition Examination Survey (NHANES), which has provided crucial insights into the health status of the US population since its first survey in 1971.^6^ Over time, NHANES expanded into an examination of a multitude of health conditions. Unfortunately, the NHANES survey process has failed to meet our current needs of timely, large-scale integrated health care data. The NHANES survey process relies on data from several thousand volunteers selected annually from 15 of the more than 3,000 counties in the United States. The five major components include a household questionnaire, a medical history questionnaire, dietary questionnaires, a physical examination by a physician, and special clinical procedures and tests, including x-rays and clinical laboratory testing. In addition, the CDC also regularly collates data provided by public health agencies, generally of varying quality and completeness and with sometimes long delays (years) prior to report release.

Despite improvements, NHANES fails to meet our current needs for timely, large-scale, integrated health care data. The burden for participant selection and the onus of participation tend to bias the sample and the observations. The nutritional assessments have long been criticized as imperfect.^7^ Timeliness is a vital consideration. Presentation of NHANES data years after their generation does not support the goals of the CDC revitalization. For illustrative examples, publication of pediatric lead poisoning by the CDC exemplifies the deficiencies with the current CDC and NHANES reporting processes. The CDC compiles pediatric blood lead reports from state and territorial health departments that may be incomplete and delayed. The most recent state data presented by the CDC cover only 28 states and the District of Columbia from 2012 to 2018 and 10 additional states with 2018 data.^8^ The CDC 2021 recommended blood lead reference level is based on the 97.5th percentile of the blood lead values among US children aged 1 through 5 years from the 2015 to 2016 and 2017 to 2018 NHANES cycles. Yet the most recent publication of NHANES pediatric lead (2021) data extends only through 2016.^9^ The 2011 to 2016 NHANES data included only 2,321 valid blood lead level results or fewer than 400 per year. Thus, the current CDC blood lead reference value is based on the equivalent of just 58 children, with results above this value including children tested as early as 2015.

A better system for collection and timely publication of such blood lead data would be to use clinical data sets produced by our clinical laboratories. As an example, a study of pediatric lead included more than 1 million results from all 50 states and the District of Columbia and overlaid several social determinants of health such as old housing stock, poverty rates, and race/ethnicity.^10^ Although only the shortcoming for timely blood lead result reporting by national public health agencies is highlighted here, additional examples abound that shine light on the flaws in continuing to employ historical NHANES processes as a keystone for public health reporting and policy recommendations. Large health care organizations, including Aetna CVS Health, Cerner, Epic, HealthVerity, IQIVA, Kaiser Permanente, LabCorp, Optum, and Quest Diagnostics, have access to many billions of health care datapoints annually from most Americans. The data are current and can be integrated with a vast amount of health care and non–health care information to allow for timely and effective reporting. Early deviations from expected patterns may offer the canaries in the coal mine, timely signals of improvements or deterioration in health or of emerging infections. Key performance metrics may be used to quickly allocate funds where most needed. Extensive efforts such as the Systemic Harmonization and Interoperability Enhancement for Laboratory Data collaborative, led by the US Food and Drug Administration and involving many other parties, are needed to normalize the data for use.^11^ Indeed, all clinical laboratories generate useful data that could be transmitted securely and efficiently to provide insights reflecting patterns of disease and disease risk for every community in the country.

The core of such a system exists. Public health reporting through the Association of Public Health Laboratories AIMS (APHL Informatics Messaging System) facilitates the secure and efficient exchange of public health data from clinical laboratories to public health agencies and among public health agencies.^12^ Among its achievements is aggregating influenza test results from public health laboratories to the CDC and biological threat data from laboratories within the laboratory response network to the CDC. This platform can be expanded to enable all clinical laboratories to provide timely data to public health agencies and, where permitted, to academics for research of public health issues.

In summary, the CDC should develop partnerships with large health care organizations with enormous coverage of health care data and other complementary non–health care data sources. And ultimately, all clinical laboratories should participate. With appropriate privacy protections, integrating these data with data such as on living conditions, in near real time, can provide timely insights into emerging infections, pockets of disease and modifiable disease risks (including insufficient vaccination rates), trends in our health, and evaluations of the effectiveness of interventions to improve population health. The CDC’s mission is “serving the public through timely action and impact.”^1^ Let’s modernize public health care data.

## Notes

Disclosure: Harvey W. Kaufman is an employee of and owns stock in Quest Diagnostics.
